# Application of Challenging Learning Based on Human-Computer Interaction under Machine Vision in Vocational Undergraduate Colleges

**DOI:** 10.1155/2022/4667387

**Published:** 2022-10-11

**Authors:** Bin Hu, Xueqiong Hong

**Affiliations:** ^1^Nanchang Business College of Jiangxi Agricultural University, Gongqingcheng 332020, Jiangxi, China; ^2^Anyang University, Anyang-si 14028, Gyeonggi-do, Republic of Korea; ^3^Gongqing College of Nanchang University, Gongqingcheng 332020, Jiangxi, China

## Abstract

Science and technology have progressed in recent years, the deepening of talent education, a challenging learning method of human-computer interaction has gradually emerged. Human-Computer Interaction (HCI for short) is the communication and interaction between humans and machines. This essay aims to apply the challenging learning combined with HCI to vocational undergraduate colleges. The GMM (that is Gaussian mixture model algorithm, commonly used in image recognition or speech recognition, etc.) algorithm is proposed in this essay to recognize students' actions. The effect of HCI is achieved by feeding back the recognized actions to the system. This essay selects 200 students from a vocational undergraduate college for challenging learning (that is, a comprehensive teaching method aiming at students' autonomous learning by stimulating students' interest in learning). The challenging learning designed in this essay is divided into 15 weeks, the task chain contains a total of 196 tasks, and the learning time is 138 h. This essay analyzes the application effects of liberal arts, male and female students, and different grades. The results show that the overall average completion rates of learning tasks for freshman, sophomore, and junior students are about 70%, 75%, and 85%, respectively, and the overall average scores for challenging learning are about 70, 78, and 83. The overall completion rate of weekly tasks for boys and girls is about 68% and 70%, and the overall average score is about 70 points and 75 points. The weekly task completion rate of liberal arts students is generally above 75%, and the overall average score is about 70 points. The overall completion rate of science students is less than 75%, and most of the learning scores are higher than 75 points. In addition, the average accuracy of the GMM algorithm for face and gesture recognition is 90% and 87%. The average frequency of students using HCI is about 320 times a day; the average score of students' experience effect of HCI is about 80 points. It may be stated that the HCI demanding learning strategy proposed in this study worked well and has achieved satisfactory learning results in the application of vocational undergraduate colleges.

## 1. Introduction

With the gradual deepening of the national talent training plan, the educational methods of vocational undergraduate colleges have also begun to innovate. At present, a challenging learning focusing on cultivating students' abilities is emerging. Challenging learning refers to students' active participation and creativity in challenging thematic learning activities inspired by the real world, emphasizing the development of higher-order thinking, the application of skills, and the internalization of concepts. Challenging learning combines a variety of learning concepts and step-by-step learning methods and conducts periodic learning with goals and tasks. The proposal of challenging learning methods will change the traditional learning methods. The traditional learning method is single, and basically one-to-many undifferentiated learning is carried out according to the course content. The purpose of learning overemphasizes the acquisition of knowledge, ignoring the cultivation of students' learning style, learning attitude, comprehensive ability, and comprehensive quality. This will lead to students lack of independent thinking, self-directed learning habits, and thinking. The learning ability of each student is different, and teachers cannot adjust the curriculum according to the learning effect of different students. HCI is specialized in studying the way of communication between humans and machines. Combining HCI for challenging learning can make students more interesting in the learning process. It can cultivate different students' comprehensive abilities such as cognitive ability, learning ability, and cooperation ability and play a role in promoting the national talent training plan. Therefore, this essay applies the challenging learning combined with HCI technology to vocational undergraduate colleges, which is of great value and significance.

Regarding the application of challenging learning, some scholars have conducted related research. Among them, Jeffries et al. examined challenging learning strategies in visually impaired (VI) adults. He recognized the importance of engineering and policy education, as well as the relationship between visually impaired mobility demands and new urban initiatives to encourage green mobility [1]. Dillette and Sipe suggested a four-year framework for challenging learning in the context of hospitality and tourist management program leader development. He outlined distinct high-impact events and results that correspond to the development of undergraduate leadership [2]. LA are learning assistants that are used to help students understand content more deeply and are particularly effective in active learning instruction. A fundamental pillar of the LA model is the LA Teaching Curriculum, which teaches LA about evidence-based teaching and how students learn. Purtell et al. used situations in which LA might manifest and reflect in the classroom to provide demanding interactive learning for LA attending pedagogy courses [3]. Puente and Kroesen compared typical college-level courses to information retention and transfer inside a 2nd undergraduate challenging test project. The results showed that students who took the design-based learning program had higher median scores on assignments than students who took the traditional course. The findings of this study serve as a suitable example of the use of design-based learning by higher education practitioners to promote retention and transfer [4]. However, these methods failed to stimulate students' interest in learning, and the learning effect was not high. It is necessary to explore high-quality methods.

To address the issues with students' lack of learning interest and low learning efficiency, some scholars have proposed methods using human-computer interaction. Among them: El Said reports thematic analysis of semistructured interviews with university students in 52 smart learner interfaces [5]. Ciechanowski et al. studied interpersonal contact, concentrating on users' emotional responses to various sorts of devices they engage with. Understanding the user side, according to Ciechanowski et al., might be crucial in building improved automation throughout the next. As a result, it can contribute to the advancement of the field of HCI [6]. Long and Aleven thought that intelligent tutoring systems (ITS) and human-computer interactive educational games assist learning by doing, and he assessed students' formula-solving abilities [7]. Chakraborty's et al. research investigated the main limitations of vision-based gesture recognition that arise in detection and preprocessing, representation and feature extraction, and recognition and explored current challenges in detail [8]. Although these methods have improved students' interest in learning to a certain extent, the actual effect is still not very satisfactory, so it needs to be further improved.

This essay adopts the GMM algorithm of HCI to study the challenging learning and proposes a challenging learning method based on HCI. This essay uses machine vision technology to obtain students' action images, and then uses the GMM algorithm and Human 3.6 M action database to recognize the images and feeds the recognition content to the system to realize HCI. This essay picks 200 individuals at random from a college for challenging learning tests. The findings indicate that the challenging learning approach proposed in this essay will have different application effects for different grades, arts and sciences, and between male and female students, but all of them can achieve good learning effects. In addition, the GMM algorithm has a high recognition effect, and this essay's HCI system received a positive rating from the students.

## 2. HCI-Based Challenging Learning Approach

### 2.1. Challenging Learning

**Figure 1:**
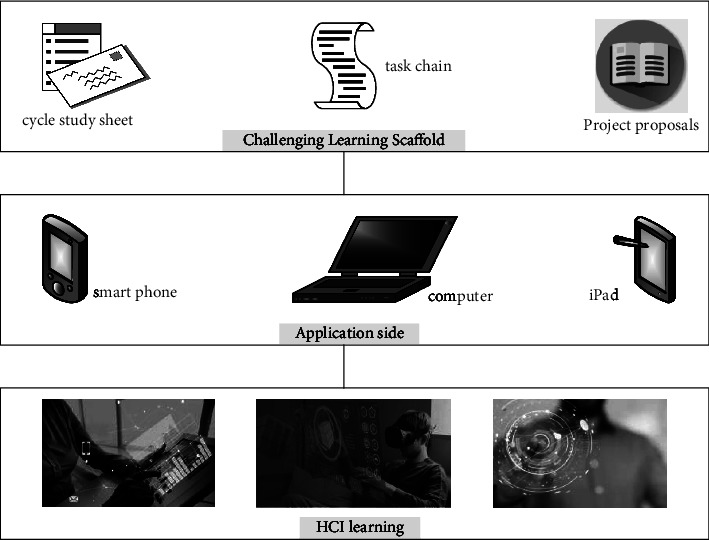
HCI-based challenging learning framework.

Challenging learning is a type of heuristic learning, which is a step-by-step learning strategy that helps to improve students' thinking and learning ability, etc. [9].  Figure 1 depicts the HCI challenge learning framework developed in this essay, which consists of a challenge learning scaffold, an application layer, and an HCI layer. The challenge learning scaffold consists of recurrent learning sheets, task chains, and project books [10]. The task chain is the deconstruction of the entire project and the superposition of various related tasks, while the cyclic learning alone is the learning of challenging tasks with time regularity. A project book usually refers to a topic of study and a research project, during which students progress through the learning process, culminating in a challenging learning master project assignment. The application layer is to develop the challenging learning scaffold into a learning software or website through a programming language so that students can learn through mobile phones, computers, tablets, and other devices [11]. This article uses java programming to build a learning website so that students can learn anytime and anywhere on the website. The HCI layer is the design of the way of communication between humans and machines. During the learning process, students interact with machines to make learning more fun [12].

The traditional challenging learning is just to build a simple challenging learning scaffold, and teachers use the learning scaffold to challenge students to learn. Although this method can stimulate students' interest in learning to a certain extent, the teaching method is still in the form of one-to-many teachers, which cannot carry out differentiated teaching, and the students' self-challenging learning efficiency is not high [13, 14]. The HCI challenging learning method designed in this essay can carry out differentiated teaching according to the different learning abilities of students and can further stimulate students' interest in learning. However, the current challenging learning's understanding of “learning” is still relatively narrow and limited, and it is more inclined to the cognitive level of psychology, and students' value, attitude, emotion, and spiritual learning are ignored [15, 16]. The reason for challenging learning is to allow students to use familiar technology and take practical problems related to students as the starting point and foothold in today's era of rapid development of information technology, which can stimulate students' learning fun and tap their potential [17, 18].

### 2.2. Design of HCI

**Figure 2:**
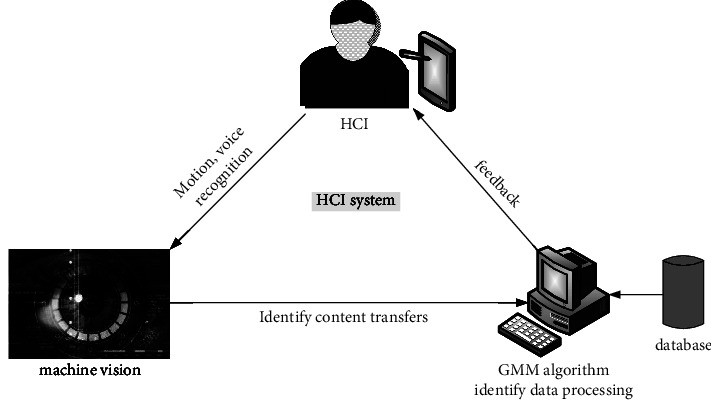
HCI system design under machine vision.

HCI is the interaction between human and machine. The design of HCI should be optimized from human touch, vision, and hearing so that users can get a more comfortable experience effect [19].  Figure 2 depicts the HCI system developed in this essay using a robotic system. During the learning process of students, the camera under machine vision collects information such as students' actions, behavior, and speech. The computer receives this information and converts it into data for processing. In this essay, the GMM method is utilized to identify and process data. The GMM algorithm uses a probability algorithm, so a data point can belong to multiple clusters, which means that the GMM algorithm can support mixed attributes. And, then feed back to the application according to the result of the identification, so that the machine can respond accordingly, thus realizing the process of HCI.

The design of HCI should follow the principles of simplicity, beauty, and convenience, bringing fun and comfort to people. The goal of this essay's HCI system is to use machine vision to recognize human behaviors. Then, the meaning of the behavior is analyzed according to the human behavior database, and the analysis result is returned to the program, and the application side makes matching interaction information [20]. This method can identify students' behaviors in real time and give more accurate feedback information in the challenging learning process.

### 2.3. GMM Algorithm

The GMM algorithm is an algorithm for unsupervised learning using a Gaussian model (a probability model based on normal distribution, often used in data analysis and image processing). In this essay, the GMM algorithm is used to identify students' action behaviors in challenging learning, so as to achieve the effect of HCI. The algorithm is identified as follows.

Assuming that the image is I, its pixels are represented by a Gaussian model, and its single initial model is

(1) $$\begin{array}{c} \left\{\begin{array}{c} \mu \left(c,g,0\right)=I\left(c,g,0\right)\\ \sigma^{2}\left(c,g,0\right)=std_{init^{2}}\text{,}\\ \sigma \left(c,g,0\right)=std_{init}\text{.} \end{array}\right. \end{array}$$

Among them, *μ* is the mean, the deviation is denoted by *ρ*, and *ρ*^2^ is the variance.

The output image is represented by Iop, then the detection result is

(2) $$\begin{array}{c} Iop\left(x,y,t\right)=\left\{\begin{array}{cc} 0\text{,} & \left|I\left(c,g,t\right)-\mu \left(c,g,t\right)\right.\left|<\lambda \times \sigma \left(c,g,t-1\right)\right.\\ 1\text{,} & otherwise \end{array}\right.\text{,} \end{array}$$

Among them, *λ* is the adjustment parameter.

With the change of time, the input and output images need to be updated, and the update method is as follows:

(3) $$\begin{array}{c} \left\{\begin{array}{c} \mu \left(c,g,t\right)=\left(1-a\right)\times \mu \left(c,g,t-1\right)+a\times \mu \left(c,g,t\right)\text{,}\\ \sigma^{2}\left(c,g,t\right)=\left(1-a\right)\times \sigma^{2}\left(c,g,t-1\right)+a\times {\left|I\left(c,g,t\right)-\mu \left(c,g,t\right)\right|}^{2}\text{,}\\ \sigma \left(c,g,t\right)=\sqrt{\sigma^{2}\left(c,g,t\right)}\text{,} \end{array}\right. \end{array}$$

Among them, *a* is the update rate.

The recognition accuracy and speed of a single model are insufficient and often cannot meet the inspection requirements. Therefore, multiple Gaussian models are often used for calculation. For the mixed Gaussian model,

(4) $$\begin{array}{c} P\left(p\right)=\left[\omega_{i}\left(c,g,t\right),\mu_{i}\left(c,g,t\right),\sigma_{i}{\left(c,g,t\right)}^{2}\right]\text{.} \end{array}$$

Among them, *ω*_*i*_(*c*, *g*, *t*) is the weight of either model, and

(5) $$\begin{array}{c} \overset{K}{\underset{i=1}{\sum }}\omega_{i}\left(x,y,t\right)=1\text{.} \end{array}$$

Among them: *K* is the number of models.

If *d* is used to represent the weight increment, it is

(6) $$\begin{array}{c} d\omega =a\times \left(1-\omega_{i}\left(c,g,t-1\right)\right)\text{.} \end{array}$$

Then, the weighted weight is

(7) $$\begin{array}{c} \omega_{i}\left(c,g,t\right)=\omega_{i}\left(c,g,t-1\right)+d\omega \text{.} \end{array}$$

Normalize the weights (The purpose of normalization is to facilitate comparison calculations and make the calculated values simpler):

(8) $$\begin{array}{c} \omega_{i}\left(c,g,t\right)=\frac{\omega_{i}\left(c,g,t\right)}{\overset{K}{\underset{i=1}{\sum }}\omega_{i}\left(c,g,t\right)}\text{.} \end{array}$$

Use the ratio of weight to standard deviation as the basis for model ranking, namely,

(9) $$\begin{array}{c} sort_{key}=\frac{\omega_{i}\left(c,g,t\right)}{\sigma_{i}\left(c,g,t\right)}\text{.} \end{array}$$

From this, the image recognition result of the mixture Gaussian model can be obtained.

### 2.4. Summary of Data Sources

The development language used in this article is java, because this programming language is relatively simple, widely used, and has strong compatibility. The development software is OpenCV, because the software contains java language interface, and the software's visual processing algorithm is powerful and the processing speed is very fast. The visual recognition database is Human 3.6 M, which contains more than 3 million three-dimensional human poses, which can meet the recognition requirements of this essay. In addition, this essay randomly selects 200 students from a vocational undergraduate college for challenging learning. The selected students include freshmen, sophomores, juniors, and students from different majors. Table 1 depicts the specific number [21].

In addition, the challenging learning designed in this essay is divided into 15 weeks, the task chain contains a total of 196 tasks, and the learning time is 138 h. As shown in Table 2, in order to allow students to gradually adapt to this learning rhythm, only 7 tasks and 7 study hours are arranged in the first and second weeks, and the average study time for one task is one hour [22, 23]. Then, the sum of tasks gradually increases, the learning time increases, and the average learning time for each task decreases.

## 3. Results and Discussion

### 3.1. Application Deconstruction of Students of Different Grades

**Figure 3:**
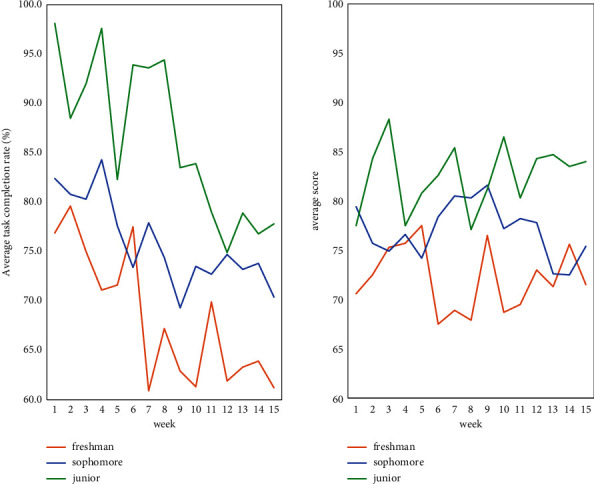
Application effect of students in different grades.

Through these 15 weeks of challenging learning, this essay makes statistics based on the students' task completion rate and teachers' scores on students' completion effects, as shown in Figure 3.

From Figure 3, it can be seen that the completion rate of freshman students is between 60% and 80%, the overall average completion rate is less than 70%, the learning score is 68–78 points, and the overall average score is about 70 points. Sophomores have a 70% to 85% completion rate, with an overall average completion rate of about 75%, and a learning score between 72 and 82, with an overall average score of about 78. Completion rates for juniors ranged from 75% to 98%, with an overall average completion rate of over 85%, with ratings ranging from 78 to 88, with an overall average rating of 83. This shows that challenging learning will be more effective for students in higher grades. This may be because students in the upper grades are more pressured to study and are more willing to take the time to study.

### 3.2. Application Deconstruction of Male and Female Classmates

**Figure 4:**
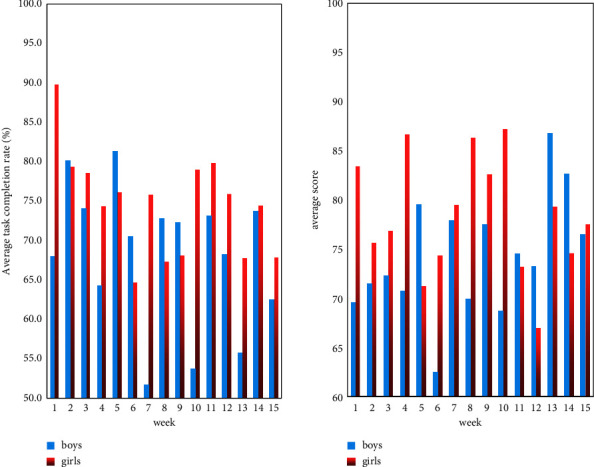
The application effect of boys and girls.

This essay does data analysis upon this proposal of male and female students to reflect the application influence of various things, as shown in Figure 4.

As can be seen from Figure 4, the weekly task completion of girls is basically higher than that of boys. The highest completion rate is 90%, the lowest is about 64%, and the overall completion rate is above 70%. The highest learning score is 87 points, the lowest is 67 points, and the overall average score is about 75 points. The weekly completion rate of boys is between 52% and 82%, and the overall completion rate does not exceed 68%. The reason for this may be that boys are more playful and spend less time on learning. Boys scored between 63 and 86 on learning, with an overall average of around 70.

### 3.3. Applied Deconstruction of Arts and Sciences

**Figure 5:**
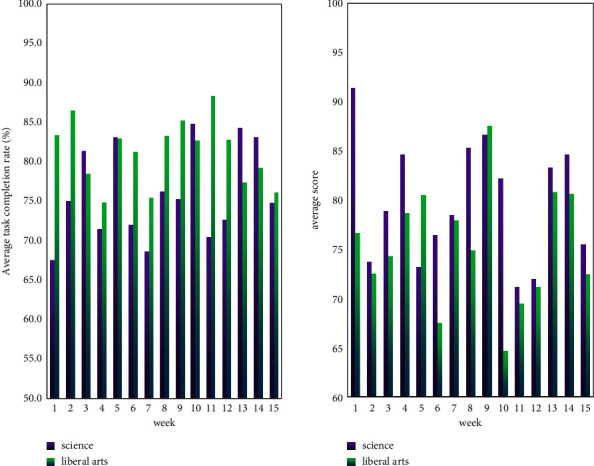
Application effect of arts and sciences.

In addition, this essay divides students into liberal arts majors and science majors according to different majors and conducts data analysis on these two categories, as shown in Figure 5.

It can be seen from Figure 5 that most of the weekly tasks in the liberal arts are better than those in the sciences, and the completion rate is generally above 75%. However, the score of learning is not as high as that of science, with scores ranging from 65 to 88, and the overall average score is about 70 points. The weekly completion rate of science students was between 65% and 84%, and the overall completion rate was lower than 75%; but most of the learning scores were higher than 75 points. It can be seen that, compared with science students, challenging learning can attract more interest in liberal arts students, but the learning effect is actually not as good as that of science students. The reason may be that liberal arts students are more willing to spend time studying, but their thinking ability is not as flexible as that of science students, so the learning effect is not as good as that of science students.

### 3.4. System Performance Deconstruction for Applications

**Figure 6:**
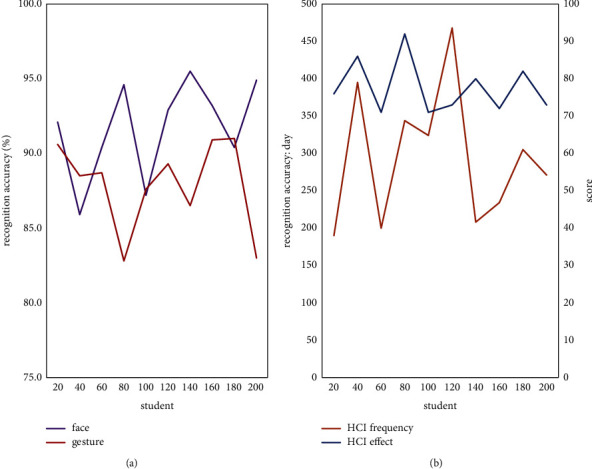
Performance analysis of the system application. (a)The recognition effect of GMM algorithm. (b) The use effect of HCI.

Finally, this essay collects the recognition results of students' faces and gestures according to the GMM algorithm during the challenging learning process and counts the frequency of students' HCI and the response effect of HCI. Among them, the response effect of HCI is that students score according to their own experience and feelings, and the full score is calculated as 100 points. The results are shown in Figure 6.

It can be seen from Figure 6 that the recognition accuracy of the GMM algorithm for faces is between 86% and 96%, and the average accuracy rate is higher than 90%. The recognition accuracy of gestures will be slightly lower, between 83% and 92%, and the average accuracy is about 87%. The recognition effect is not bad, but there is still room for improvement. In addition, students use HCI at a maximum of 480 times a day and a minimum of 230 times, with an average frequency of about 320 times. The students' scores for HCI experience are between 70 and 90 points, and the average score is about 80 points, which shows that the HCI method designed in this essay can be loved by most students and can achieve good practical application results.

## 4. Conclusion

As a new way of learning, challenging learning can stimulate students' interest in learning and improve learning efficiency, so it should be applied to vocational undergraduate colleges as soon as possible. Over the years, with said advancement of device vision technology, people have found HCI to be an efficient and interesting way to interact. Therefore, this essay first studies the specific content of challenging learning and HCI and finds that the key to challenging learning is to build a challenging learning scaffold, and the design of the HCI system requires the use of multiple recognition techniques. Then, this essay proposes a GMM recognition algorithm, applies it to the recognition of HCI systems and constructs a challenging learning system based on HCI. Secondly, the learning system was tested. From the grades, genders, and completion of challenging learning in the arts and sciences, it was known that the students' completion of learning tasks and scores were good. From the system performance test, it is concluded that the recognition accuracy of the GMM algorithm has become more sophisticated, and the students' perception of the use of HCI has become more complex. Therefore, the challenging learning system designed in this essay has obtained decent results in the application of vocational undergraduate colleges. However, the authors acknowledge that their capabilities are limited, and the research has much room for improvement. The author's research on HCI is insufficient, the experiment is not perfect, and the author still needs to conduct more in-depth research and exploration and strive to improve in future work.

## Figures and Tables

**Table 1:** Students involved in challenging learning.

Gender	Boys	Girls
Freshman	46	32
Sophomore	49	30
Junior	25	18
Total	120	80

**Table 2:** Challenging learning arrangements.

Period	Number of tasks	Total study duration:h	The average duration of the task:h
Week 1	7	7	1.00
Week 2	7	7	1.00
Week 3	8	7.5	0.94
Week 4	8	7.5	0.94
Week 5	10	8	0.80
Week 6	10	8	0.80
Week 7	12	9	0.75
Week 8	12	9	0.75
Week 9	15	10	0.67
Week 10	15	10	0.67
Week 11	15	10	0.67
Week 12	19	11	0.58
Week 13	19	11	0.58
Week 14	19	11	0.58
Week 15	20	12	0.60
Total	196	138	0.70

## Data Availability

The data used to support the findings of this study are available from the corresponding author upon request.
